# 

**Published:** 1878-12

**Authors:** 


Whole Number, CCCCIII. **December, 1878.** Number 6, Vol. XXXVII.

### THE CHICAGO MEDICAL Journal and Examiner

**EDITORS:**

WILLIAM H. BYFORD, A.M., M.D.,
JAS. NEVINS HYDE, A.M., M.D. FERD. C. HOTZ, M.D.
E. FLETCHER INGALS, M.D.

Chicago Medical Press Association. Aug. 2D 1875.

CHICAGO:
*PUBLISHED BY THE CHICAGO MEDICAL PRESS ASSOCIATION.*
188 Clark Street.

☞Read the Notice of this Journal among Advertisements.

